# Voxelwise atlas rating for computer assisted diagnosis: Application to congenital heart diseases of the great arteries

**DOI:** 10.1016/j.media.2015.09.001

**Published:** 2015-12

**Authors:** Maria A. Zuluaga, Ninon Burgos, Alex F. Mendelson, Andrew M. Taylor, Sébastien Ourselin

**Affiliations:** a Translational Imaging Group, Centre for Medical Image Computing, University College London, UK; b Centre for Cardiovascular Imaging, UCL Institute of Cardiovascular Science, London, UK; c Cardiorespiratory Division, Great Ormond Street Hospital for Children, London, UK

**Keywords:** Computer-aided diagnosis, Voxel rating, Image synthesis, Atlases, Congenital heart diseases

## Abstract

Atlas-based analysis methods rely on the morphological similarity between the atlas and target images, and on the availability of labelled images. Problems can arise when the deformations introduced by pathologies affect the similarity between the atlas and a patient’s image. The aim of this work is to exploit the morphological dissimilarities between atlas databases and pathological images to diagnose the underlying clinical condition, while avoiding the dependence on labelled images. We propose a voxelwise atlas rating approach (VoxAR) relying on multiple atlas databases, each representing a particular condition. Using a local image similarity measure to assess the morphological similarity between the atlas and target images, a rating map displaying for each voxel the condition of the atlases most similar to the target is defined. The final diagnosis is established by assigning the condition of the database the most represented in the rating map.

We applied the method to diagnose three different conditions associated with dextro-transposition of the great arteries, a congenital heart disease. The proposed approach outperforms other state-of-the-art methods using annotated images, with an accuracy of 97.3% when evaluated on a set of 60 whole heart MR images containing healthy and pathological subjects using cross validation.

## Highlights

• This paper presents a voxelwise atlas rating approach for computer-aided diagnosis.
• The method relies on multiple atlas databases, but does not require annotated images.
• The method reports an accuracy of 97.3%, which is higher than other state-of-the-art methods.

## Graphical abstract

**Figure:**
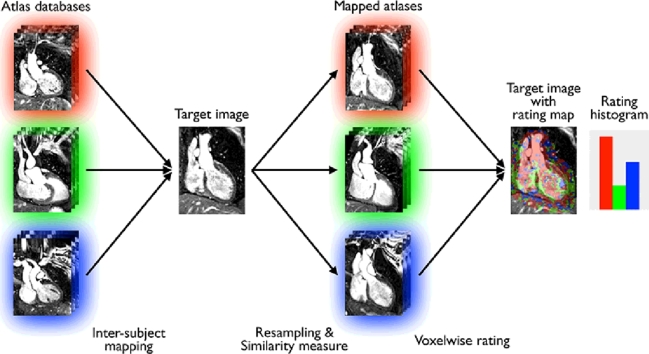

## 1 Introduction

In the last years, atlas-based techniques (Rohlfing et al., 2004) have become a well established method for medical image segmentation. In this approach, intensity images within an atlas database are first registered to an unseen or target image. The obtained transformations are used to map the set of atlases into the target image space and, once transformed, the label images from each atlas are combined into a final consensus segmentation by applying a fusion criterion.

Despite its widespread use, atlas-based segmentation remains to have two major drawbacks. First, the morphological similarity between the atlases and the target image must be guaranteed. When this condition is not satisfied, the deformations induced by the morphological dissimilarities may affect the atlas registrations, leading to unrepresentative segmentations. This situation is common when pathologies introduce morphological alterations in the affected organs. Second, atlas-based segmentation relies on the availability and quality of labelled images. In a clinical scenario or research project with limited resources, gathering a sufficient amount of good quality annotated images of pathology-specific, or even healthy subjects, is a challenge.

Recent research has tried to address the problem of morphological dissimilarity in several ways. Based on the segmentation stage that they tackle, we classify existing methods into four categories: atlas construction, atlas or model selection, image registration and segmentation analysis. A common characteristic of all these families of methods is that they do not solve the dependence on labelled images.

Methods that fall into the atlas construction category try to minimise the errors produced by image dissimilarity by optimally constructing a single atlas, from the set of atlases, that is the most representative of the population (Fischl, Salat, Busa, Albert, Dieterich, Haselgrove, van der Kouwe, Killiany, Kennedy, Klaveness, Montillo, Makris, Rosen, Dale., 2002, Guimond, Meunier, Thirion, 2000, Joshi, Davis, Jomier, Gerig, 2004). To account for the variations introduced by pathologies, the constructed atlas can include probabilistic map (Lorenzo-Valdes et al., 2004) to provide spatially and temporally varying a priori information.

Model selection techniques aim to select an on-the-fly atlas subset that fits best the target image, and remove potential outliers that affect the final segmentation. Results have shown that it performs better than single atlases in different applications (Hammers, Allom, Koepp, Free, Myers, Lemieux, Mitchell, Brooks, Duncan, 2003, van Rikxoort, Isgum, Arzhaeva, Staring, Klein, Viergever, Pluim, van Ginneken, 2010, Zuluaga, Cardoso, Modat, Ourselin, 2013). As a consequence, model selection approaches have gained popularity and several research groups have explored automatic model selection techniques. Typically, these techniques address atlas selection by performing an atlas ranking via a global (Aljabar, Heckemann, Hammers, Hajnal, Rueckert, 2009, Leung, Barnes, Ridgway, Bartlett, Clarkson, Macdonald, Schuff, Fox, Ourselin, 2010), a local criterion (Cardoso, Leung, Modat, Keihaninejad, Cash, Barnes, Fox, Ourselin, 2013, Išgum, Staring, Rutten, Prokop, Viergever, van Ginneken., 2009) or a combination of both (van Rikxoort et al., 2010).

More recently, a novel method has been proposed in which the problem of morphological dissimilarity is addressed at the image registration stage. The proposed approach makes use of low-rank image decomposition to formulate a registration framework capable of handling images containing large pathologies and large deformations (Liu et al., 2014). Although this is a promising alternative, the method still needs to be better evaluated on datasets with ground truth tissue labels (Liu et al., 2014).

The last family of algorithms, denoted here as segmentation analysis methods, analyses the results obtained through the use of different atlases to improve the accuracy of the final segmentation (Hanna, Barschdorf, Klinder, Weber, Krueger, Dössel, Lorenz, 2011, Kutra, Saalbach, Lehmann, Groth, Dries, Krueger, Dössel, Weese, 2012) or to exploit the information obtained from the analysis within the context of computer-aided diagnosis (CAD) (Zuluaga et al., 2014).

In this work, instead of trying to minimise the errors induced by morphological dissimilarities, we exploit the variations introduced by pathologies. Following the principle developed by Zuluaga et al. (2014) that an unrepresentative atlas is more likely to lead to a poor segmentation, we assume that an atlas will be morphologically more similar to the target when both share the same clinical condition. The method, called voxelwise atlas rating (VoxAR), exploits morphological dissimilarities between atlas and target images at the local scale. This method relies on multiple atlas databases representing different conditions and does not require labelled images. VoxAR uses a local image similarity measure to assess the morphological similarity between atlas and target images. A rating map displaying for each voxel the condition of the atlas most similar to the target is then defined. As in Zuluaga et al. (2014), the proposed method is used within the context of CAD and, more specifically, CAD of congenital heart diseases. For this purpose, a diagnosis is established by assigning the condition of the database with the highest number of occurrences in the rating map.

This paper is organised as follows: Section 2 provides an overview of CAD methods for cardiovascular diseases, motivates the selection of an atlas-based approach for this task and describes the data to be used. The proposed method, VoxAR, is detailed in Section 3. Section 4 describes the experiments that were performed and the obtained results. Section 5 discusses the results and, finally, Section 6 presents the conclusions.

## 2 Clinical context

### 2.1 Computer-aided diagnosis in cardiovascular diseases

The use of computer aided diagnosis in cardiovascular diseases has not been as widely used as in other areas (Chen, Suzuki, MacMahon, 2011, Drukker, Giger, Metz, 2005, Summers, Beaulieu, Pusanik, Malley, Jeffrey, Glazer, Napel, 2000). As in other domains, pathology must be defined in how it deviates from normality. Methods can be classified into two groups based on how differences are detected: those which represent images as a set of extracted features (Teßmann, Vega-Higuera, Fritz, Scheuering, Greiner, 2009, Zuluaga, Hush, Delgado Leyton, Hernández Hoyos, Orkisz, 2011a, Zuluaga, Magnin, Hernández Hoyos, Delgado Leyton, Lozano, Orkisz, 2011b), and those which compare them to a model or atlas built using a healthy population (Lorenzo-Valdes, Sanchez-Ortiz, Elkington, Mohiaddin, Rueckert, 2004, Zhang, Wahle, Johnson, Scholz, Sonka, 2010, Duchateau, Craene, Piella, Silva, Doltra, Sitges, Bijnens, Frangi, 2011, Lombaert, Peyrat, Croisille, Rapacchi, Fanton, Cheriet, Clarysse, Magnin, Delingette, Ayache, 2012). The first group of methods has been applied mainly to the diagnosis of ischemic heart disease, whereas the second has been used to identify abnormalities in ventricular volumes (Lorenzo-Valdes et al., 2004), cardiac motion (Duchateau et al., 2011), myocardial fibre orientation (Lombaert et al., 2012) and congenital heart disease (Zhang et al., 2010). A common limitation in both groups is that methods can only differentiate between normal and abnormal, without providing further information on the pathology.

Atlas-based methods have been successful in cardiac CAD due to the morphological alterations that pathologies introduce in the heart. These cause registration errors that can be exploited to provide insight into how a pathology deviates from normality. This concept was first used by van Rikxoort et al. (2010) in a segmentation framework capable of selecting the atlas closest to the target image under the rationale that the most similar atlas to the target image is expected to provide the best segmentation. Zuluaga et al. (2014) extended this idea in the context of cardiac CAD. Where atlas sets with different morphologies are available, it is possible to segment an image with the different sets and then use the measured quality of the obtained segmentations to determine which of them provides the best fit. It is assumed that the best fitting atlas share the same clinical condition as the target.

### 2.2 Dextro-transposition of the great arteries

Congenital heart disease (CHD) is a broad term which refers to abnormalities of the cardiac structure and function caused by abnormal or disordered heart development before birth (Zhang, 2007). Although a lifetime follow-up is usually required, it has been shown that not enough physicians have a specialised training for the number of adult CHD patients (Gurvitz et al., 2005). For this reason, it is common that patients are lost to follow-up, and can be examined by clinicians who have no experience of imaging CHD. Under such scenario, semi or fully automated methods that can assist non-experts in diagnosis are highly desired. The morphological alterations caused by the abnormal heart development make suitable the usage of atlas-based methods for their diagnosis.

**Fig. 1:**
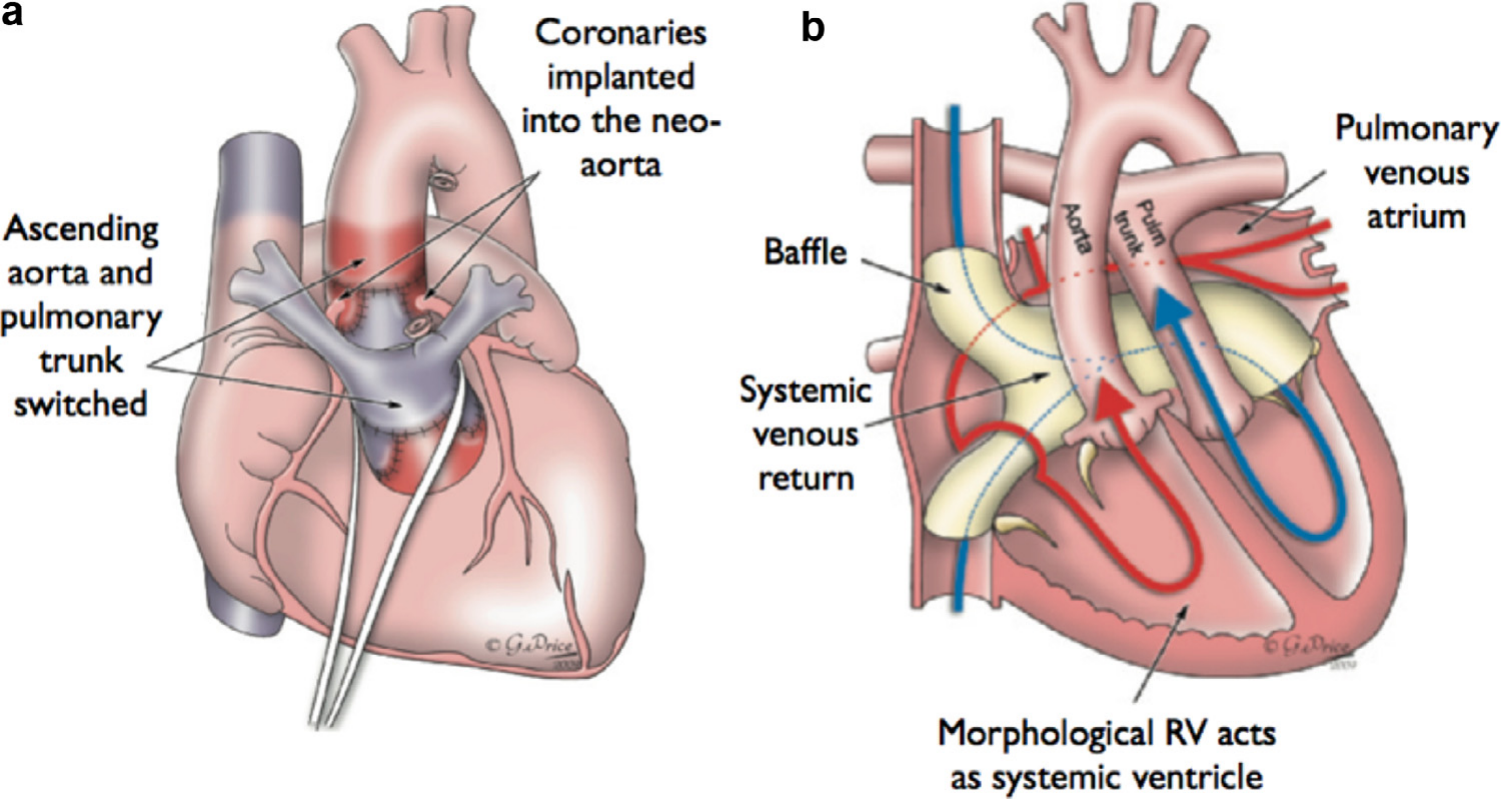
(a) Schematic drawing of Arterial Switch operation showing Le Compte manoeuvre with the translocation of aortic and pulmonary arteries. (b) Schematic drawing of an atrial switch (Mustard/Senning) for transposition of the great arteries. Systemic (blue) blood is directed from the superior caval vein and inferior caval vein into the left atrium, then via the mitral valve to the left ventricle and then to the pulmonary artery. Pulmonary (red blood) is directed from the pulmonary veins to the right atrium, then via the tricuspid valve to the aorta. Images produced with permission from Gemma Price. (For interpretation of the references to colour in this figure legend, the reader is referred to the web version of this article.)

The clinical focus of this study is the diagnosis of dextro-transposition of the great arteries (d-TGA). This condition is a congenital heart defect in which the two major vessels that carry blood away from the heart, *i.e.* the aorta and the pulmonary artery, are switched. Neonates born with d-TGA require surgery immediately after birth. There are two different procedures to repair d-TGA at birth: arterial switch (ASO) and atrial switch operation (Senning or Mustard procedure). In ASO, which is the most common procedure, the arteries are switched to their usual positions, i.e. the pulmonary artery arising from the right ventricle and the aorta from the left ventricle, and the coronary arteries are reattached to the aorta. In atrial switch operation, the arteries are left in place, but a baffle is created between the top chambers of the heart, allowing oxygen-poor blood to move from the right atrium to the left ventricle and out the pulmonary artery to the lungs. Returning oxygen-rich blood moves through the baffle from the left atrium to the right ventricle and out the aorta (Fig. 1).

As it is common with CHDs, a characteristic of both ASO and atrial switch is that the “normal” morphology of the heart is not recovered. Monitoring of the patient across their lifetime is crucial as infants who have these surgeries are not cured and might have lifelong complications.

### 2.3 Data

The identification of the anatomical variations caused by the correcting procedures is a complex task which requires high clinical expertise. Although echocardiography tends to be the modality of choice in CHD diagnosis, it has shown limited value for the assessment of the great vessels (Fenchel et al., 2006). Cardiovascular MR (CMR) has proven to provide a reliable and accurate assessment of CHD (Hirsch, Kilner, Connelly, Redington, Sutton, Somerville, 1994, Fenchel, Greil, Martirosian, Kramer, Schick, Claussen, Sieverding, Miller, 2006). Nevertheless, diagnosis remains challenging, especially in children and young adults (Fenchel et al., 2006), and it is often missed during screening.

We acquired 3D, electrocardiography- and respiratory-gated CMR images from 60 cases – 20 anatomically normal hearts and 40 patients with d-TGA who had undergone either an ASO (20), or an atrial switch operation (20). All imaging was done as part of routine clinical practice. The study had institutional approval, and all patients (and/or their parents or guardian) gave informed consent for the use of their data for research purposes.

CMR imaging was performed using a 3D whole-heart MR angiography sequence. The images, covering the entire heart, were obtained in a sagittal orientation by using a magnetisation-prepared, 3D balanced, steady-state free precession sequence (TR 3.0 ms, TE 1.5 ms, flip angle 90°, number of lines per segment acquired per cardiac cycle 30−40, sensitivity-encoding factor 2.0, bandwidth per pixel 590 Hz, field of view 280 × 280 × 120 mm^3^, acquisition matrix 192 × 192 × 80 and iso-volumetric voxel size 1.5 × 1.5 × 1.5 mm^3^). In all cases, 3D whole-heart imaging was performed 5–10 min after the administration of routine gadolinium contrast agent.

## 3 Method

Atlas-based methods involving a registration step are sensitive to morphological differences between the atlas and target subjects. Rather than trying to reduce the errors created by these dissimilarities, the proposed method exploits the variations introduced by pathologies and assumes that an atlas will be more similar to the patient’s image when both atlas and target share the same clinical condition. The developed methodology makes use of pre-acquired databases of images, each database comprising subjects presenting a defined pathology. The morphological similarity between atlas and target images is assessed by computing a local similarity measure.

**Fig. 2:**
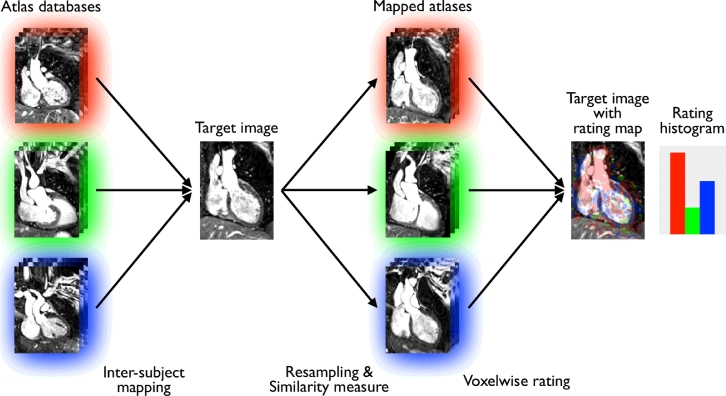
VoxAR: voxelwise atlas rating for computer-aided diagnosis. All the atlases from each database presenting a clinical condition (red, green, and blue) are registered to the target image. A local image similarity measure is computed between the mapped atlases and the target image and then ranked across all atlases and databases. The diagnosis corresponds to the condition the most represented in the rating map, which displays for each voxel the condition of the atlases the most similar to the target. Finally, the rating histogram displays the percentage of occurrences of each condition. (For interpretation of the references to colour in this figure legend, the reader is referred to the web version of this article.)

Here, instead of considering the databases separately, as done in Zuluaga et al. (2015), we directly compare the atlases from all the databases to the target. We then define a rating map displaying for each voxel the condition of the atlases the most similar to the target. The final diagnosis is established by assigning the condition of the database the most represented in the rating map. A diagram illustrating our voxel atlas rating approach (VoxAR) is shown in Fig. 2

### 3.1 Preprocessing: mask definition and inter-subject mapping

Given a set of clinical conditions *Ω*, an atlas database $D_{\omega }={\left\{J_{n}^{\omega },M_{n}^{\omega }\right\}}_{n=1}^{N\omega }$ is defined as the paired set of *N_ω_* MR images sharing condition *ω* ∈ *Ω*, $J_{n}^{\omega },$ and associated image masks $M_{n}^{\omega }$ containing the heart. As opposed to standard atlas-based methods, these masks are not required to be highly precise. They can therefore be obtained through simple segmentation techniques and morphological operations. More particularly, they are obtained via Otsu thresholding, connected component analysis and a set of morphological operations.

Let *I* be the target image to be diagnosed. The proposed method requires the alignment of the *N_ω_* MR images from each atlas database *D_ω_* to the target. Registration of cardiac images is challenging due to the structures surrounding the heart (e.g. ribs, liver) that tend to bias the registration. To avoid such problem, we divide the registration into two stages. At the first stage, we seek to define a region of interest (ROI) that encloses the heart in *I* and removes the surrounding structures. The registration restricted to the defined ROI comes as a second stage.

The first step to obtain the registration ROI for the unseen image is to affinely register (Ourselin et al., 2001) *I* to the intensity images of each atlas database. The obtained transformations are applied to the mask images of each database, which are then fused using a simple majority voting criterion. As a result, a binary mask roughly englobing the heart of the target image is obtained as an output.

After the affine alignment step, a non-rigid free form deformation registration (Modat, Ridgway, Taylor, Lehmann, Barnes, Fox, Hawkes, Ourselin, 2010, Rueckert, Sonoda, Hayes, Hill, Leach, Hawkes, 1999) using normalised mutual information (Studholme et al., 1999) is applied to align the atlases with the unseen image. To avoid the bias that surrounding structures can produce in the registration, the unseen image is masked using the binary mask obtained during the affine stage.

This inter-subject mapping setup had been previously validated using the Niftyreg package (Zuluaga et al., 2013). A list of the parameters used is summarised in Table 1. The Niftyreg documentation further details the parameters configured in the registration package^2^.

After non-rigidly transforming the atlases to the unseen image space, the obtained transformations are used to map the *N_ω_* MRI/mask pairs of each atlas database *D_ω_* to the target image. A refined location of the heart is achieved by fusing the *N_ω_* mapped masks of an atlas database *D_ω_* and thresholding the probabilistic output of majority voting (threshold of 0.8). The resulting mask for the condition *ω* is denoted *M_ω_*.

Finally, to assure that the different atlas databases contain the same amount of information, we define for each target subject a ROI *M*, which corresponds to the intersection of each database mask

(1) $$M=\underset{\omega \in \Omega }{⋂}M_{\omega }.$$

In the following, the processing steps are restricted to this ROI.

### 3.2 Previous work: Image Synthesis Approach (ISA)

We first formulate our previous work, called Image Synthesis Approach (ISA) (Zuluaga et al., 2015) as a reference method in CAD using a global measurement to exploit the differences in morphology induced by different pathologies. Similarly to Burgos et al. (2014), the ISA consists of fusing the mapped atlases according to their morphological similarity to the target in order to create a synthetic image for each condition represented in the databases. The final diagnosis is established by assigning the condition whose synthetic image is most similar to the true image as measured by a global similarity measure.

#### 3.2.1 Image/morphological similarity

The morphological similarity between the target image and the set of registered atlases is assessed using a local image similarity measure, the local normalised correlation coefficient (LNCC). This measure evaluates the quality of alignment between two images by calculating the correlation between the signals. The convolution-based LNCC, as implemented by Cachier et al. (2003), between the target image *I* and the *n^th^* mapped image of atlas database *D_ω_*, $J_{n}^{\omega },$ at voxel *x*, is given by:

(2) $${\text{LNCC}}_{n}^{\omega }\left(x\right)=\frac{\langle I\left(x\right),J_{n}^{\omega }\left(x\right)\rangle }{\sigma \left(I\left(x\right)\right)\;\sigma \left(J_{n}^{\omega }\left(x\right)\right)}.$$

The means and standard deviations at voxel *x* are calculated using a Gaussian kernel $G_{\sigma_{G}},$ with standard deviation $\sigma_{G}=2$ voxels, through convolution:

$$\begin{array}{ccc} \bar{I(x)} & = & G_{\sigma_{G}}*I\left(x\right),\;\sigma \left(I\left(x\right)\right)=\sqrt{\bar{I{\left(x\right)}^{2}}-{\bar{I(x)}}^{2}},\\ \langle I(x),J(x)\rangle & = & \bar{I(x)\cdot J(x)}-\bar{I(x)}\cdot \bar{J(x)}, \end{array}$$

where * denotes the convolution operator. We chose a smaller Gaussian kernel than the one used by Burgos et al. (2014) ($\sigma_{G}=3$ voxels) to capture smaller differences between the images.

#### 3.2.2 Intensity fusion

The LNCC at each voxel is ranked across all atlas images in the database *D_ω_* and the ranks, noted as $r_{n}^{\omega }\left(x\right),$ are converted to weights by applying an exponential decay function:

(3) $$\alpha_{n}^{\omega }\left(x\right)=e^{-\beta r_{n}^{\omega }\left(x\right)}$$

with $\alpha_{n}^{\omega }\left(x\right)$ being the weight associated with the nth atlas image at voxel *x* and β=0.5 (Burgos et al., 2014).

As suggested by Cardoso et al. (2012), the final synthetic MR image is obtained by a spatially varying weighted averaging process. As the MRI intensity scale is not standardised, the images are normalised prior to the fusion step. We used a standard score normalisation:

$$I{\left(x\right)}_{norm}=\frac{I\left(x\right)-\bar{I_{M}}}{\sigma (I_{M})}$$

where the mean and standard deviation are computed within *M* (Eq. (1)).

The weights $\alpha_{n}^{\omega }\left(x\right)$ are used to reconstruct the synthetic MR image *I^ω^* at voxel *x* ∈ *M* as follows:

(4) $$I^{\omega }\left(x\right)=\frac{\sum_{n=1}^{N_{\omega }}\alpha_{n}^{\omega }\left(x\right)\cdot J_{n}^{\omega }\left(x\right)}{\sum_{n=1}^{N_{\omega }}\alpha_{n}^{\omega }\left(x\right)}.$$

#### 3.2.3 Global ranking and final diagnosis

Synthetic images are generated for each pathology represented in the databases. Each synthetic image *I^ω^* is then compared to the target image *I* using the global normalised correlation coefficient

(5) $$NCC^{\omega }=\frac{\langle I,I^{\omega }\rangle }{\sigma \left(I\right)\;\sigma \left(I^{\omega }\right)}.$$

Following the idea that morphological similarity can be measured through image similarity, the synthetic images are ranked based on the NCC score. A final diagnosis is established by assigning the condition *ω* of the top-ranked synthetic image *I^ω^* to the target image.

### 3.3 Voxelwise atlas rating (VoxAR)

Similarly to the Image Synthesis Approach, VoxAR assumes that the atlases which share the same condition as the patient being diagnosed will be morphologically more similar to the target image than the atlases presenting another pathology. In this approach, the local similarity measure is directly exploited to establish the diagnosis.

Once mapped to the target subject, the atlas images are rated according to their morphological similarity when compared to the target. To assess this similarity, the LNCC is computed between all the atlases from each database and the target.

#### 3.3.1 Rating map construction

Instead of considering the databases independently as in the ISA approach, the atlases most similar to the target are obtained by ranking the LNCC across the atlas images from all the databases. The ranks are then used to define a rating map *R*.

Given $L_{n}^{\omega }\left(x\right),$ a binary variable equal to 1 if the rank $r_{n}^{\omega }\left(x\right)$ is among the T-top atlases at voxel *x*, the number of atlases from a particular condition *ω* which are among the T-top ranked can be expressed as

(6) $$A^{\omega }\left(x\right)=|{\left\{r_{n}^{\omega }\left(x\right)\cdot L_{n}^{\omega }\left(x\right)\right\}}_{n=1}^{N_{\omega }}|,$$

where | · | denotes the cardinality operator. *T* controls the number of atlases to be evaluated for the construction of *R*. Using *A^ω^*(*x*), the rating map *R* at *x* is equal to the condition of the atlas most present among the T-top most similar to the target

(7) $$R\left(x\right)=\left\{ \begin{array}{cc} \underset{\omega \in \Omega }{arg max}\left(A^{\omega }\left(x\right)\right), & \text{if}\;A^{\omega^{*}}\left(x\right)\geq \left\lceil \frac{T}{2}+1\right\rceil .\\ \infty , & \text{otherwise}. \end{array}\right.$$

with $\omega^{*}={arg max}_{\Omega }\left(A^{\omega }\left(x\right)\right)$. The condition $A^{\omega^{*}}\left(x\right)\geq \left\lceil \frac{T}{2}+1\right\rceil$ guarantees that only the atlas condition that represents the absolute majority among the T-top ranked will be assigned to *R*(*x*).

#### 3.3.2 Final diagnosis

From the rating map, we define a rating histogram by computing the percentage of occurrences of each condition *ω* within *R*. Only the voxels with a non-infinite value are considered to compute the histogram. The diagnosis corresponds to the pathology of the database with the highest number of occurrences. The rating histogram can also be used as a measure of confidence by assessing the separation between each bin.

## 4 Evaluation and analysis

In order to assess the clinical utility of the proposed CAD system, we tested its capability to identify the surgical procedure used to treat a congenital heart disease, the d-TGA (Section 2). Three groups of images were used as atlas databases *D_ω_*, ω∈Ω={ASO, Atrial, Normal}, each database counting $N_{\omega }=20$ subjects. The number of atlases considered when constructing the rating map was set to T=7.

### 4.1 Comparison with other methods

At a first instance, we compared the performance of VoxAR with two other approaches. First, we considered the Image Synthesis Approach (ISA) (Zuluaga et al., 2015), which is the seminal idea behind the formulation of VoxAR but, through the use of a global measure. Afterwards, we analysed the performance of our proposed framework w.r.t. a state-of-the-art segmentation-based approach (SBA) (Zuluaga et al., 2014). For a fair comparison with both methods, we have followed the evaluation protocols described in the original publications (Zuluaga, Mendelson, Cardoso, Taylor, Ourselin, 2014, Zuluaga, Burgos, Taylor, Ourselin, 2015). In the following, further details are provided for each of them.

#### 4.1.1 VoxAR vs. ISA

**Fig. 3:**
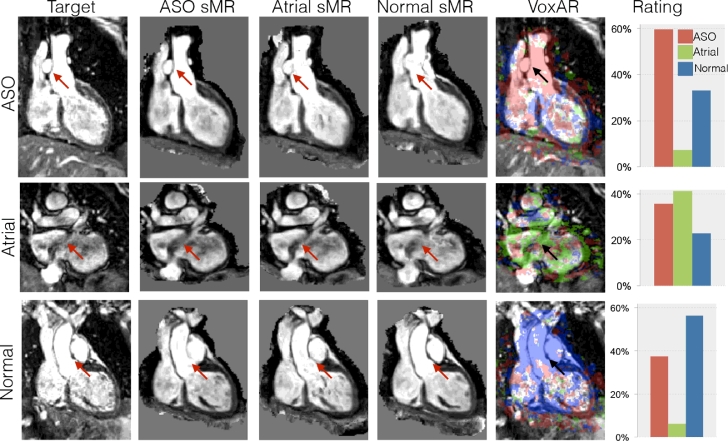
Diagnosis examples. For every target (first column), synthetic MR images (sMR) are obtained using three atlas databases, ASO, atrial and normal, with ISA. Arrows point to areas where morphological differences between the atlas databases and the target are prone to produce anatomically inconsistent images. The fourth column displays the target image with the rating map obtained through VoxAR, followed by the corresponding rating histogram.

We first compared VoxAR with ISA (Zuluaga et al., 2015), in order to discover if a local similarity measure (VoxAR) would provide a more reliable diagnosis than a global one (ISA). The comparison was performed using the maximum possible of atlases per database within a leave-one-out cross validation scheme. The obtained confusion matrices are displayed in Table 2, and examples of diagnosed images through both approaches are shown in Fig. 3.

Results show that both VoxAR and ISA have a 100% sensitivity for d-TGA, as they are capable of discriminating pathological from healthy subjects: no pathological cases were diagnosed as healthy. However, VoxAR appears to have a higher accuracy when identifying the specific underlying pathology. It has a higher precision (90% instead of 75%) when identifying the atrial condition. The overall error rate of VoxAR (5%) is lower than that one of ISA (10%), indicating the better performance of the local-based similarity measure.

**Fig. 4:**
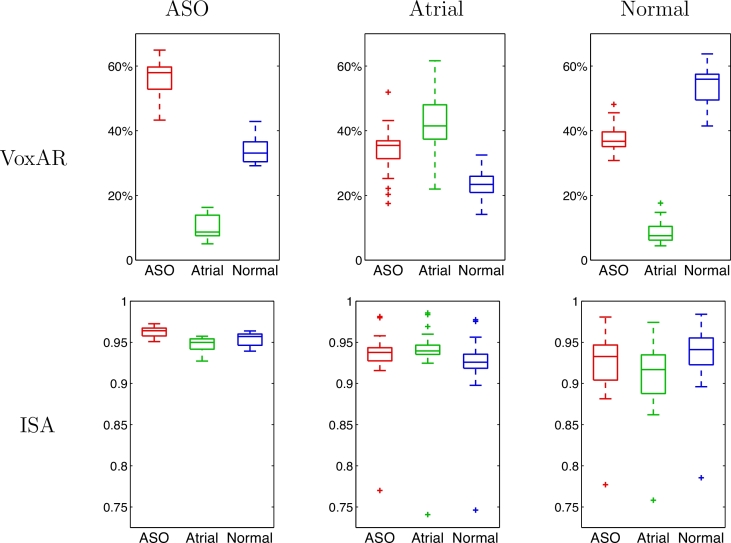
Boxplots displaying the median, lower and upper quartiles, minimum and maximum of the percentage of voxels (top) originating from each database and the NCC (bottom) for the ASO (left), atrial (centre) and normal (right) target conditions.

For further analysis, we plotted the percentage of voxels originating from each database for ASO, Atrial and Normal target images, and the NCC computed between every target image and the respective synthetic images (Fig. 4). The displayed results show that, for VoxAR, the distributions for a measure (pertaining to a single database) for each condition are well separated, as are the distributions for each measure across subjects of single condition. This is not the case for the NCC measures.

### 4.2 VoxAR vs. SBA

The segmentation-based CAD framework proposed by Zuluaga et al. (2014) builds on the hypothesis that well and poorly segmented images have different distributions in some representative feature space, making it possible to discriminate them. As the use of an unrepresentative atlas is likely to lead to a poor segmentation, this discrimination tells about the morphological similarity between an unseen image and an atlas database. The method extracts a set of features describing the quality of a segmentation (obtained through multi-atlas segmentation), and introduces them into a logical decision tree that provides the final diagnosis.

For evaluation, we used the same atlases as reported by Zuluaga et al. (2014). These databases consisted of a set of 5 annotated images for each clinical condition, acquired at the same centre, and containing labels of the four main chambers, the myocardium, the aorta and the pulmonary artery.

Table 3 reports the sensitivity, specificity, positive predictive value (PPV) and negative predictive value (NPV) of VoxAR and SBA in discriminating pathological vs. non-pathological subjects. Accuracy measures the capacity of both methods to identify each specific condition. For the sake of completeness, ISA results are also reported. When only 5 atlases for each condition are available, SBA outperforms both ISA and VoxAR.

### 4.3 Sensitivity to atlas database size

**Fig. 5:**
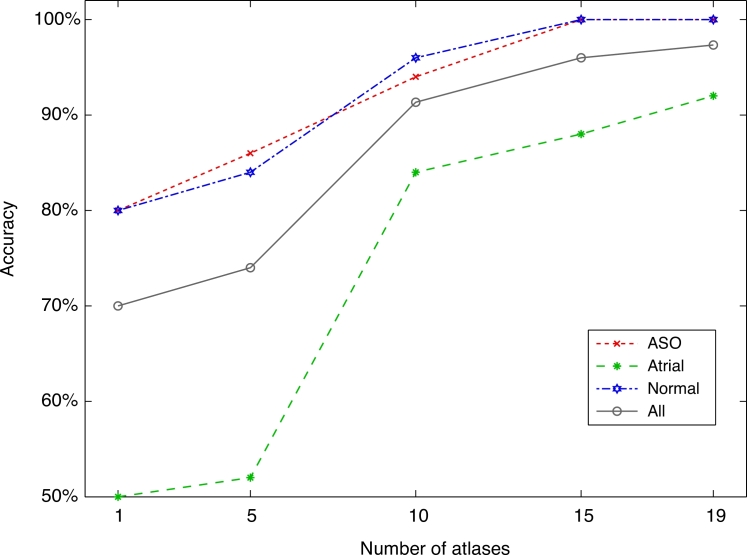
Performance of VoxAR relative to the number of atlases in each database.

After assessing the better performance of VoxAR, we focused on evaluating how changes in the size of the atlas databases could hamper VoxAR’s performance. For this task, we used a cross validation scheme that is a generalisation of balanced repeated K-fold cross validation (see Appendix). Fig. 5 plots the mean accuracy of VoxAR as a function of the number of atlases in each database (L=50 repeats for each experiment). Results show that the system’s performance improves with databases size, reaching a maximum accuracy of 97.3% when $N_{\omega }=19$. The CAD system failed to diagnose atrial condition in only 4 out of 50 trials, where the patients were flagged as ASO. However, it reports 100% sensitivity as it was able to identify all patients with a congenital heart disease condition. Normal condition was successfully diagnosed reporting a specificity of 100%.

### 4.4 Sensitivity to unbalanced atlas database sizes

To further study the influence of the atlas databases on the proposed method, we investigated its sensitivity to unbalanced database sizes.

The experiment consisted of studying two settings:

(a) the size of the target pathology’s database is less than those of the other pathologies; and
(b) the size of the target pathology’s database is greater than those of the other pathologies.

In the first setting, the number of atlases originating from the atlas database corresponding to the target pathology, the “correct” database, was fixed to 10. The size of the two remaining databases was set to 15 and 15 (50% difference), 15 or 19 (70% difference), and 19 and 19 (90% difference). In the second scenario, the “correct” atlas database consisted of 19 atlases and the size of the two remaining databases was set to 15 and 15 (50% difference), 15 or 10 (70% difference), and 10 and 10 (90% difference). For both settings, each experiment was repeated 10 times (L=10).

**Fig. 6:**
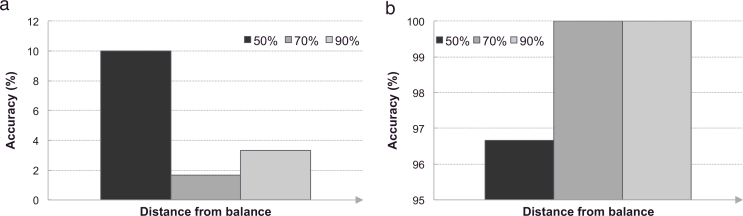
Bar plots displaying the sensitivity of the proposed method to unbalanced atlas database sizes. (a) When the size of the database corresponding to the target pathology is less than the size of the other databases, the diagnosis is biased towards the other databases. (b) When the size of the database corresponding to the target pathology is superior to the size of the other databases, the diagnosis is biased towards the pathology of the target.

Results indicate that unbalanced atlases introduce a bias towards the dominant database. As a consequence, when the number of atlases in the “correct” database is less than the number of atlases in the other databases, the diagnosis is biased towards the other databases (Fig. 6(a)) leading to poor accuracies. The diagnosis accuracy for balanced databases of 10 atlases is of 91.3% (Fig. 5) and decreases to 10.0%, 1.7% and 3.3% when the distance to balance increases by 50%, 70% and 90%, respectively. Conversely, when the number of atlases in the database corresponding to the target pathology is greater than the number of atlases in the other databases, the diagnosis is biased towards the “correct” database (Fig. 6(b)). We note that this bias appears when the difference is large (at least 70%). For balanced databases of 19 atlases, the diagnosis accuracy is of 97.3% (Fig. 5). A moderate change (50%) only affects the accuracy (96.7%) slightly. However, the accuracy increases to 100% when the distance compared to balance goes to 70% and 90%.

### 4.5 Performance in the presence of missing pathologies

A potential drawback of the proposed CAD framework is its behaviour in the presence of missing or unknown pathologies, *i.e.* pathologies that are not considered among the *Ω* conditions with an associated atlas database *D_ω_*. To assess the behaviour of the method when a missing pathology is to be diagnosed, we applied it to four CMR images of patients with tetralogy of Fallot (TOF), another form of congenital heart disease. The CAD system diagnosed all the evaluated images as ASO. Naturally, the CAD system could not identify TOF as it is absent from the atlas databases; however, all four patients were correctly flagged as pathological.

**Fig. 7:**
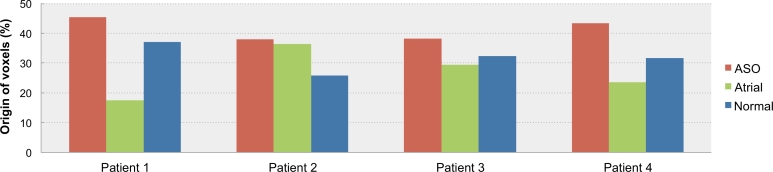
Rating histograms from each atlas database when diagnosing four patients with tetralogy of Fallot, a pathology not represented in the databases.

To further analyse the response of our algorithm to unknown pathologies, we have plotted the rating histograms, presenting the percentage of voxels originating from each database, when diagnosing the four patients (Fig. 7). The amounts of voxels originating from each of the atlas databases are close to each other, although one of the pathological databases does consistently dominate. This behaviour shows that the CAD system is not as certain in its response as when a known pathology is diagnosed.

## 5 Discussion

In order to avoid the use of labels within the diagnosis pipeline, we have developed a computer-aided diagnosis framework, denoted voxelwise atlas rating (VoxAR), based on multiple atlas databases that uses the variations introduced by pathologies to identify the underlying clinical condition of a subject. The presented framework builds over the hypothesis that an atlas will be morphologically more similar to a target image when both share the same condition. VoxAR is based on our previous approach, denoted ISA, in which synthetic images are regenerated from each atlas database trying to reproduce the target. The diagnosis was established by comparing the synthetic images to the target through a global measure. Conversely to ISA, VoxAR uses a local measure to assess the morphological similarity between the atlases and target: a rating map in which each voxel contains information about the most similar condition at the specific location. The diagnosis is established by constructing a histogram of the rating map and selecting the condition of the database with the highest frequency.

The performance of the CAD framework was assessed and evaluated on a set of 60 whole heart MR images containing healthy and pathological subjects with two variations of d-TGA, a congenital heart disease. Using a leave-one-out cross validation, we first compared VoxAR and ISA. We found that, if both methods have a 100% sensitivity for d-TGA, VoxAR has a higher accuracy when identifying the specific underlying pathology. The less accurate results obtained using ISA can be explained by the process used to generate the synthetic images. Using a ranking scheme where larger weight is given to the images better registered to the target image tends to compensate for the registration inaccuracies (Yushkevich et al., 2010) and attenuates the differences introduced by pathologies. VoxAR, by directly measuring the similarity between the target image and the registered atlases in a voxel-wise manner, avoids this error compensation.

Using a generalisation of balanced repeated K-fold cross validation, we have evaluated the method’s sensitivity to changes in the size of the atlas databases. Our results showed that the use of larger databases is encouraged as it increases the CAD’s framework performance (Fig. 5). Although this finding is not particularly new, it points out one of the advantages of VoxAR. While larger atlas databases are desirable, expanding the databases is not easily achievable when accurate labelling is needed. By not depending on labelled images, VoxAR (as well as ISA) reduces the costs of database enlargement.

We also investigated the sensitivity of the method to unbalanced databases. We showed that when the number of atlases in the database corresponding to the pathology of the target is smaller than the number of atlases in the other databases, the diagnosis is biased towards the other databases (Fig. 6(a)). Conversely, when the size of the database corresponding to the target pathology is larger than the size of the other databases, the diagnosis tends to be in favour of the “correct” database (Fig. 6(b)). Therefore, to avoid creating a bias towards a pathology, the atlas databases should be equally balanced.

The obtained results and its reasonable computational time (25 min) suggest that, in clinical practice, the CAD system could be used to assign clinical labels to screening patients without an existing label. VoxAR has reported 100% sensitivity and specificity, which implies that all patients with congenital heart disease were correctly identified. This means that if the algorithm were to be used to detect transposition of the great arteries, neither would any patient have missed the appropriate clinical management, nor any healthy subject have been referred for unnecessary further evaluation. The estimated accuracy (capability of identifying correctly the specific condition) of 97.3% is higher than the accuracies of other state-of-the-art methods following the same principle (Zuluaga et al., 2014) and of our seminal work using synthetic images (Zuluaga et al., 2015). Furthermore, the proposed approach does not require the use of labelled images and is not limited to the diagnosis of two conditions. It can be extended to any number of conditions as long as there is an associated atlas database available.

Two previous works have addressed problems in a similar fashion. Coupé et al. (2012) propose a method that simultaneously segments an image and generates a grading measure to estimate the similarity of the patch surrounding the voxel under study with all the patches present in a training population. As the training population includes data from subjects in two different clinical states, it is possible to measure the degree of closeness to one group or another for each voxel. Finally, an average grading value is computed over all the voxels of the segmented structure and thresholding is applied to provide a diagnosis. Although this method uses a principle similar to ours, it addresses a different clinical problem (Alzheimer’s disease).

Within the context of cardiovascular image analysis, van Rikxoort et al. (2010) have developed a multi-atlas segmentation framework (AMAS and ALMAS) that uses a similarity measure and a stopping criterion to perform segmentation with only the set of atlas that are closest to the target. Interestingly the method showed that, when segmenting brain images with an atlas database, only adult datasets were used to segment adult scans, pediatrics were used for pediatrics and elderly atlases for elderly target scans. This suggests that the AMAS & ALMAS framework could be used for diagnosis purposes. For instance, their atlas selection and stopping criterion have a role similar to the rating histogram of our proposed approach. Thanks to the stopping criterion, this method has the advantage of being potentially faster than ours, particularly, in the scenario of large atlas databases. It remains to validate if the diagnosis feature of AMAS and ALMAS could also be applied to pathological cardiac images.

One of the current limitations of the proposed framework is its inability to identify a condition that is not represented in an atlas database. To evaluate the behaviour of VoxAR when diagnosing pathologies not represented within the databases, we used four subjects with tetralogy of Fallot. In every case, VoxAR flagged a clinical condition (e.g. ASO) which suggests that the method is capable of differentiating pathological from non-pathological. Interestingly, the obtained rating histograms (Fig. 7) suggest that, in the presence of a missing pathology, the frequencies of each condition are very close to each other. This can be interpreted as the system being less certain of its answer than it is when the condition is “known”. Such behaviour can also occur in the presence of known pathologies (see Fig. 3, atrial case), especially when the image to be diagnosed is noisy. Based on this behaviour, a natural extension of the proposed approach would be to define a way to include the values of the rating histogram as part of the diagnosis. In this way, the system would provide a diagnosis and a confidence measure (such as posterior probability). However, these hypotheses should be validated using a larger number of subjects than the four cases currently used.

This paper has focused on congenital heart disease (CHD) diagnosis and, more specifically, in the diagnosis of d-TGA. CHDs are an ideal target for the proposed method since they introduce morphological changes in the anatomy which the registration algorithms (associated to an atlas-based framework) cannot cope with. While such morphological changes are found mostly in the heart, the use of the global (ISA) or local (VoxAR) similarity measures could serve as an indicator of the evolution of a pathology. Further experiments would be required to validate this hypothesis in other regions of the body.

## 6 Conclusion

This paper presents a local approach for atlas-based computer-aided diagnosis relying on multiple atlas databases, each representing a particular condition, which does not require annotated images. A local image similarity measure assessing the morphological similarity between the atlas and target images is used to generate a rating map displaying for each voxel the condition of the atlas most similar to the target. The diagnosis is established by assigning the condition of the database the most represented in the rating map. The results obtained with the proposed approach outperform the state-of-the-art methods using annotated images, with an accuracy of 97.3% when evaluated on a set of 60 whole heart MR images containing healthy and pathological subjects using cross validation.

## Figures and Tables

**Table 1:** Registration settings for the pre-processing step. The registration is performed in two stages: an affine step (rigid followed by affine) and a non-rigid registration.

Reg. stage	Type	Parameters
1	Rigid	Coarse-to-fine levels: 3
		Max. iterations: 5
1	Affine	Coarse-to-fine levels: 3
		Max. iterations: 8
2	Non-rigid	Coarse-to-fine levels: 3
		Max. iterations: 300
		Bending energy: 0.005

**Table 2:** Confusion matrix using VoxAR (top) and ISA (bottom). Evaluation was performed using a leave-one-out approach for a total of 20 experiments for each method.

	ASO	Atrial	Normal
ASO	1	0	0
Atrial	0.10	0.90	0
Normal	0.05	0	0.95
	ASO	Atrial	Normal

ASO	1	0	0
Atrial	0.25	0.75	0
Normal	0.05	0	0.95

**Table 3:** Sensitivity, specificity, negative predictive value (NPV), positive predictive value (PPV) and accuracy of VoxAR, ISA and SBA.

	VoxAR			ISA			SBA
Number of atlases	5	19		5	19		5
Sensitivity	90.0	100		52.5	100		100
Specificity	70.0	95.0		60.0	95.0		90.0
NPV	100	100		38.7	100		100
PPV	86.9	97.5		72.4	97.5		93.0
Accuracy	83.3	95.0		55.0	90.0		93.3

## Appendix: Validation scheme

When N=A−n subjects of each condition were to be used as atlases (per database) in *L* repeats, the *n* to be held out for testing were randomly selected in the following way: each subject was given an integer counter, initially zero, representing the number of times they had been left out so far. To select the *n* of each condition at each repeat in sequence, as many subjects as possible (up to *n*) with the minimum counter value were drawn (randomly without replacement). Then, if that number was less than *n*, subjects with the higher counter value were drawn. All subjects drawn had then their counters incremented by one. In this way, only two counter values exist at any time. Each subject has an average accuracy as measured by all the times they were included in the test set, and the accuracy in identifying a condition is given by the average of all the accuracies for all its subjects. When Lq=A/n for integer *q* and L=A/n, this is equivalent to balanced K-fold cross validation fully repeated *q* times.

## Footnotes

2 http://sourceforge.net/projects/niftyreg/ .
